# Prevalence of Asymptomatic Mpox among Men Who Have Sex with Men, Japan, January–March 2023

**DOI:** 10.3201/eid2909.230541

**Published:** 2023-09

**Authors:** Daisuke Mizushima, Yui Shintani, Misao Takano, Daisuke Shiojiri, Naokatsu Ando, Takahiro Aoki, Koji Watanabe, Takato Nakamoto, Hiroyuki Gatanaga, Shinichi Oka

**Affiliations:** National Center for Global Health and Medicine, Tokyo, Japan (D. Mizushima, Y. Shintani, M. Takano, D. Shiojiri, N. Ando, T. Aoki, K. Watanabe, T. Nakamoto, H. Gatanaga, S. Oka);; Personal Health Clinic, Tokyo (D. Shiojiri); Kumamoto University, Kumamoto, Japan (H. Gatanaga, S. Oka)

**Keywords:** Mpox, monkeypox virus, viruses, sexually transmitted infections, asymptomatic, men who have sex with men, MSM, Japan

## Abstract

We prospectively assessed asymptomatic monkeypox virus infections among men who have sex with men in Tokyo, Japan, during the initial phase of the mpox epidemic. Our findings suggest that asymptomatic infections were likely underestimated and were comparable in magnitude to symptomatic infections, highlighting the need to improve testing accessibility among high-risk populations.

During the 2022 global mpox outbreak, asymptomatic monkeypox virus (MPXV) infections or unrecognized mpox cases were reported among men who have sex with men (MSM) (*1*–*4*). Asymptomatic cases were diagnosed by using PCR on anorectal, pharyngeal, urine, and pooled samples, but the prevalence of asymptomatic infections varied by country. A recent meta-analysis reported the prevalence of asymptomatic MPXV infections worldwide (*5*), but limited cohort sizes have hindered precise estimations. Understanding the extent to which asymptomatic infections contribute to MPXV transmission is crucial for an effective public health response (*6*,*7*). In addition, further clarification on the how prevalence of asymptomatic infections affect mpox epidemics is needed. 

By December 2022, only 8 mpox cases had been confirmed in Japan, and 5 of those cases were reported in Tokyo. However, since the beginning of 2023, the number of new mpox cases has steadily increased in Japan. Despite the rise in cases, vaccination for mpox remains unavailable in Japan, even for high-risk populations, such as MSM and persons with HIV. We assessed asymptomatic MPXV infections among MSM cohorts with and without HIV infection in Tokyo.

## The Study

We assessed mpox prevalence among MSM across 3 sites in Tokyo during January 5–March 20, 2023. We enrolled MSM >18 years of age who had sexual intercourse within the previous 3 months and who provided written informed consent. We excluded persons from the study if they reported symptoms of suspected mpox at enrollment. Specifically, we excluded persons who had typical mpox symptoms, which include suspected skin lesions and any of the following: fever, lymphadenopathy, or pain in mucous membranes. We categorized atypical symptoms as having 1 typical mpox symptom, such as fever or pain, or other atypical symptoms.

Patients self-collected clinical samples for MPXV testing, including anorectal swab samples or pooled samples consisting of anorectal swabs, initial stream urine, and gargle rinse, by using a previously reported method (*8*). Persons who tested positive for MPXV were closely monitored through weekly health checks conducted via telephone and asked about their general condition, including whether they had any atypical symptoms. 

We defined asymptomatic infections as mpox cases without any symptoms, including atypical symptoms, during the study period. We classified symptomatic infections as mpox cases in which any symptoms developed, including atypical symptoms, <3 weeks before mpox testing or during the study period. Also, to increase awareness of mpox, we provided all study participants with general information on the disease, including its mode of transmission and typical symptoms. 

We used the QIAamp DNA Mini Kit (QIAGEN, https://www.qiagen.com) to extract viral DNA from patient specimens and QuantStudio 12K Flex and QuantiTect Probe PCR Kit (Thermo Fisher Scientific, https://www.thermofisher.com) to subsequently detect viral DNA. To measure the copy numbers for genomic DNA of MPXV and of varicella zoster virus, which is used as a differential diagnosis, we performed a specific multiplex quantitative PCR, as previously reported (*9*). This study was approved by the Human Research Ethics Committee of National Center for Global Health and Medicine (approval no. NCGM-S-004600-00).

**Figure 1:**
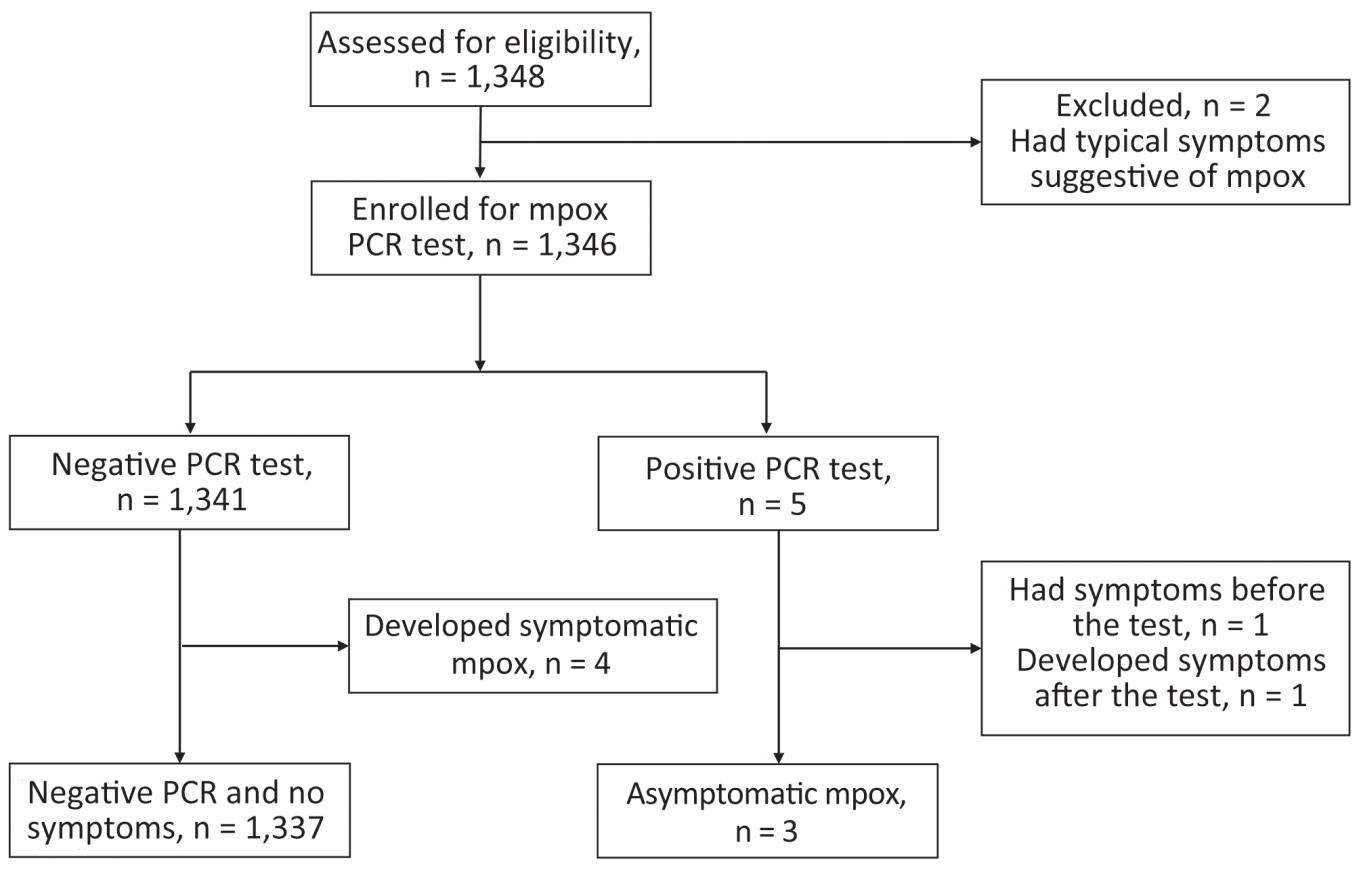
Flowchart of participant selection in a study of prevalence of asymptomatic mpox among men who have sex with men, Japan, January–March 2023. Of 1,348 eligible participants, 2 were excluded because of suggestive mpox symptoms. Of the remaining 1,346, a total of 5 tested positive for mpox via reverse transcription PCR; 4 of those who initially tested negative later had mpox symptoms develop. Ultimately, 6 cases were categorized as symptomatic monkeypox virus infections and 3 as asymptomatic. A total of 1,337 participants tested negative and did not exhibit any symptoms during the study period.

We recruited a total of 1,348 eligible MSM for this study (Figure 1; Appendix Table). Two persons were excluded because of suspected symptoms associated with mpox, which were subsequently confirmed outside of this study to be MPXV infection by PCR testing of skin lesions. The remaining 1,346 participants had a median age of 38 (IQR 31–47) years and underwent PCR testing for MPXV. Among participants, 5 (0.37%; 95% CI 0.12–0.86) tested positive. One positive result was obtained from an anorectal swab, and the remaining 4 were from pooled samples (Table; Appendix Figure 1). Of the 5 positive cases, cycle threshold values were 20.8–31.0. The time interval between last sexual activity and mpox diagnosis was 8–48 days. Three of the positive cases remained asymptomatic after 1 month and were classified as asymptomatic infections. However, 1 participant, upon receiving the positive result, disclosed recovering from fever and pharyngitis without experiencing typical skin manifestations of mpox 1 week before the study quantitative PCR test, and another participant reported having only skin lesions 3 days after the screening test. Consequently, we classified those 2 cases as symptomatic MPXV infections. 

**Table:** Characteristics among cases in a study of prevalence of asymptomatic mpox among men who have sex with men, Japan, January–March 2023*

Case no.	Classification	HIV status	Sample type	Ct value	Timeframe, d					Symptoms
					Sexual activity to diagnosis	Sexual activity to symptom onset	Negative test to symptom onset	Symptom onset to diagnosis	Negative test to diagnosis	
1	Asymptomatic	On PrEP	Anorectal swab	21.2	10	NA	NA	NA	NA	NA
2	Asymptomatic	HIV	Pooled†	20.8	29	NA	NA	NA	NA	NA
3	Asymptomatic	On PrEP	Pooled†	28.4	ND	NA	NA	NA	NA	NA
4	Symptomatic‡	HIV	Pooled†	28.8	8	11	NA	NA	NA	Skin lesions
5	Symptomatic§	On PrEP	Pooled†	31.0	48	8	NA	NA	NA	Fever and pharyngitis
6	Symptomatic¶	HIV	Skin lesion swab	ND	22	15	32	7	39	Skin lesions
7	Symptomatic¶	On PrEP	Skin lesion swab	ND	23	18	48	5	53	Skin lesions, fever, lymphadenopathy, and pharyngitis
8	Symptomatic¶	On PrEP	Skin lesion swab	ND	18	9	13	9	22	Skin lesions, fever, lymphadenopathy, and pharyngitis
9	Symptomatic¶	HIV	Skin lesion swab	ND	8	4	53	4	57	Skin lesions and lymphadenopathy

*Ct, cycle threshold; NA, not applicable; ND, no data; PrEP, preexposure prophylaxis. †Samples consisted of anorectal swabs, initial stream urine, and gargle rinse. ‡Initially asymptomatic but had symptoms develop during study. §Initially asymptomatic, but patient later reported previous self-resolving symptoms. ¶Initially tested negative but had symptoms develop during study.

**Figure 2:**
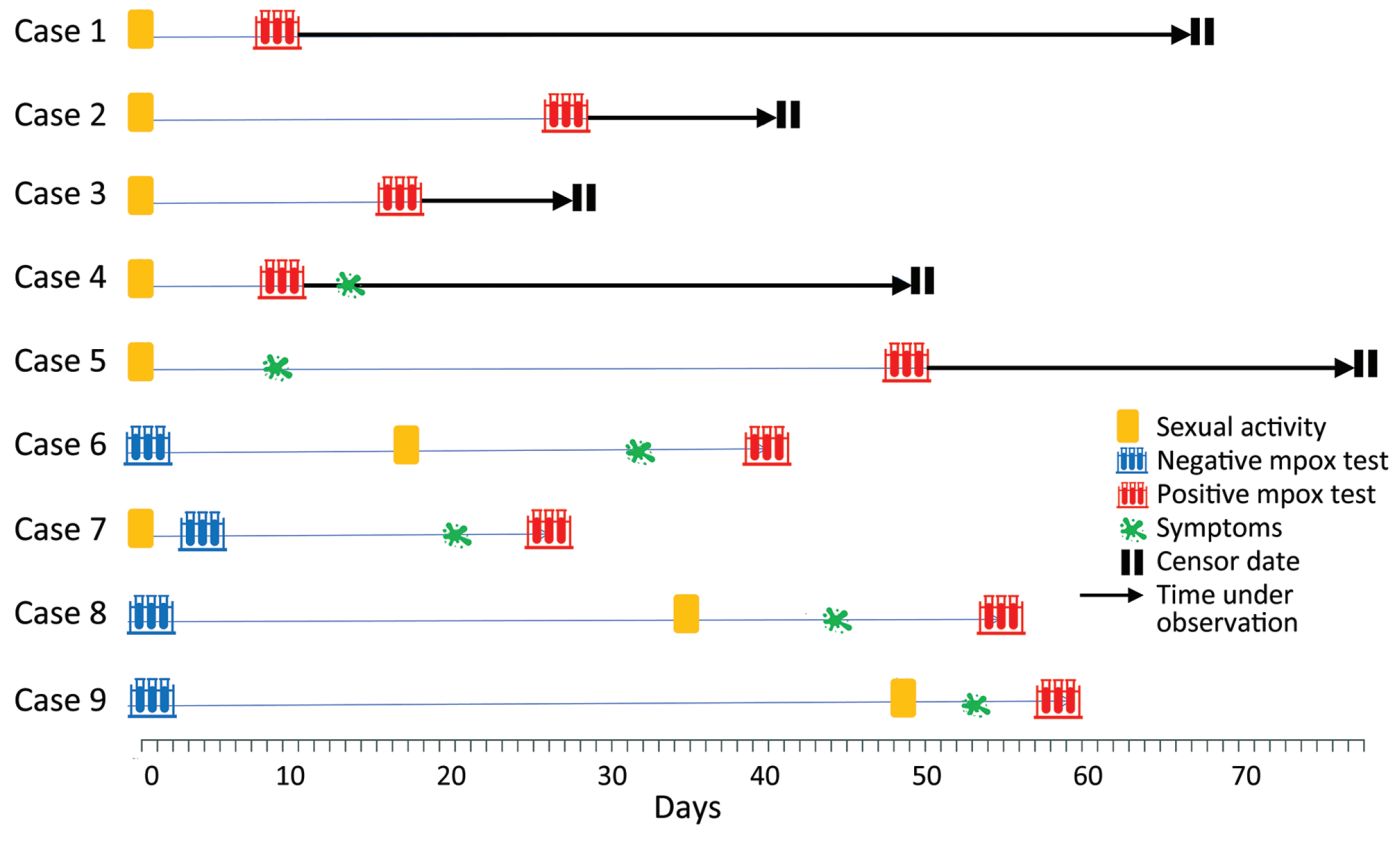
Timeline of sexual activity, symptoms, and testing for participants in a study of prevalence of asymptomatic mpox among men who have sex with men, Japan, January–March 2023. Data are provided for 9 participants who were positive for monkeypox virus during the study period. Cases 1–3 remained asymptomatic.

Of the 1,341 MSM who tested negative by PCR, 4 participants had symptoms of suspected mpox develop after the study test and were later confirmed MPXV-positive by PCR testing of skin lesions. The time from the negative PCR test to symptom onset was 13–53 days, and the time from symptom onset to diagnosis was 4–9 days (Figure 2). Of all mpox cases, 4 were MSM with HIV infection; 1 was asymptomatic and 3 were symptomatic. Those 4 case-patients were receiving antiretroviral therapy, had a CD4 count >500 cells/mL, and had an undetectable HIV viral load. During the study period, a total of 44 cases were identified in Tokyo (Appendix Figure 2).

## Conclusions

We conducted a large-scale study on mpox prevalence in Japan and found that prevalence of unrecognized or asymptomatic mpox might be underestimated. By applying a rigorous definition of asymptomatic MPXV infection, we identified a similar number of asymptomatic mpox cases compared with symptomatic cases among MSM cohorts in Tokyo, regardless of whether participants had typical or atypical mpox symptoms. These findings provide valuable insights into the intricacies of asymptomatic mpox cases and underscore the pressing need to enhance the availability of mpox testing for high-risk populations experiencing atypical symptoms. In addition, our prospective approach combined with the large cohort and timely surveillance conducted at the onset of the mpox epidemic in Japan (*10*) enabled us to determine a relatively precise prevalence of asymptomatic MPXV infection.

Although the specific infectivity of asymptomatic cases has not yet been determined, the potential prevalence of undetected asymptomatic mpox cases could contribute to the current global pandemic (*11*), which might be supported by our cycle threshold value data that was <30 in asymptomatic cases. To gain a comprehensive understanding of the infectivity of asymptomatic mpox cases, including the duration of viral shedding, further investigation is required. In addition, the lack of awareness of mpox could be affecting the prevalence of undetected cases; most participants in our study were not aware of mpox. Therefore, enhanced awareness, including knowledge of atypical symptoms, and research on infectivity are critically needed to mitigate the potential spread of MPXV. 

This study had some limitations. First, ≈70% of the subjects were tested by using only anorectal samples and cross-sectional tests at 3-month intervals, which might have underestimated of asymptomatic mpox prevalence. Second, the infectivity and duration of viral shedding were not evaluated in asymptomatic cases, thereby limiting our understanding of the role of asymptomatic persons in MPXV transmission. Finally, the study did not use MPXV antibody testing because of the low specificity of currently available modalities (*12*,*13*); thus, we might have missed additional asymptomatic mpox cases.

In conclusion, our study offers valuable insights into the relative magnitude between asymptomatic and symptomatic MPXV infection among MSM cohorts during the early stages of the mpox epidemic in Japan. Further research is needed to comprehend the epidemiology and clinical significance of asymptomatic mpox, including examination of the infectivity and the duration of viral shedding in asymptomatic cases. Nonetheless, our study highlights the urgent need for mpox awareness, testing, and vaccination among high-risk groups, including MSM and HIV-positive persons, in Japan.

Appendix Additional information on prevalence of asymptomatic mpox among men who have sex with men, Japan, January–March 2023.
