# Supplementary material for: Prevalence of Asymptomatic Mpox among Men Who Have Sex with Men, Japan, January–March 2023
Source: Emerg Infect Dis. 2023 Sep;29(9):1872–6. doi: 10.3201/eid2909.230541 (PMC10461655; doi:10.3201/eid2909.230541)
Supplement: Appendix — Additional information on prevalence of asymptomatic mpox among men who have sex with men, Japan, January–March 2023. [file 23-0541-Techapp-s1.pdf]

Article DOI: https://doi.org/10.3201/eid2909.230541

*EID cannot ensure accessibility for supplementary materials supplied by authors. Readers who have difficulty accessing supplementary content should contact the authors for assistance.*

# Prevalence of Asymptomatic Mpox among Men Who Have Sex with Men, Japan, January–March 2023

## Appendix

**Appendix Table.** Baseline characteristics of 1,346 participants in a study of prevalence of asymptomatic mpox among men who have sex with men, Japan, January–March 2023*

| Characteristics | Participants (n = 1346) |
|---|---|
| Median age, y (IQR) | 38 (31–47) |
| Median days of follow-up (IQR) | 33 (15–54) |
| Study site† | |
| Hospital A | 277 (20.6) |
| Clinic B | 150 (11.1) |
| Clinic C | 919 (68.3) |
| Bacterial infection, n = 1,313‡ | |
| Chlamydia | 131 (10) |
| Gonorrhea | 72 (5.5) |
| Any STI | 190 (14.5) |
| HIV status | |
| HIV-positive§ | 277 (20.6) |
| HIV-negative | 1,069 (79.4) |
| Sexual orientation | |
| Gay | 843 (62.6) |
| Bisexual | 90 (6.7) |
| Missing data | 413 (30.7) |
| Sexual role | |
| Insertive | 232 (17.2) |
| Receptive | 253 (18.8) |

| Characteristics | Participants (n = 1346) |
| --- | --- |
| Both | 421 (31.3) |
| Missing data | 440 (32.7) |

*Data are no. (%) unless otherwise stated. STI, sexually transmitted infection.

†Hospital A primarily serves patients with HIV infection; clinic B serves patients without HIV who are on pre-exposure prophylaxis (PrEP); and clinic C primarily serves men who have sex with men and are using PrEP.

‡Data on bacterial infections were missing from 33 participants; this analysis included 1,313 participants and each infection was verified by testing during the study.

§All participants with HIV were on anti-retrovirus therapy and had stable viral suppression.

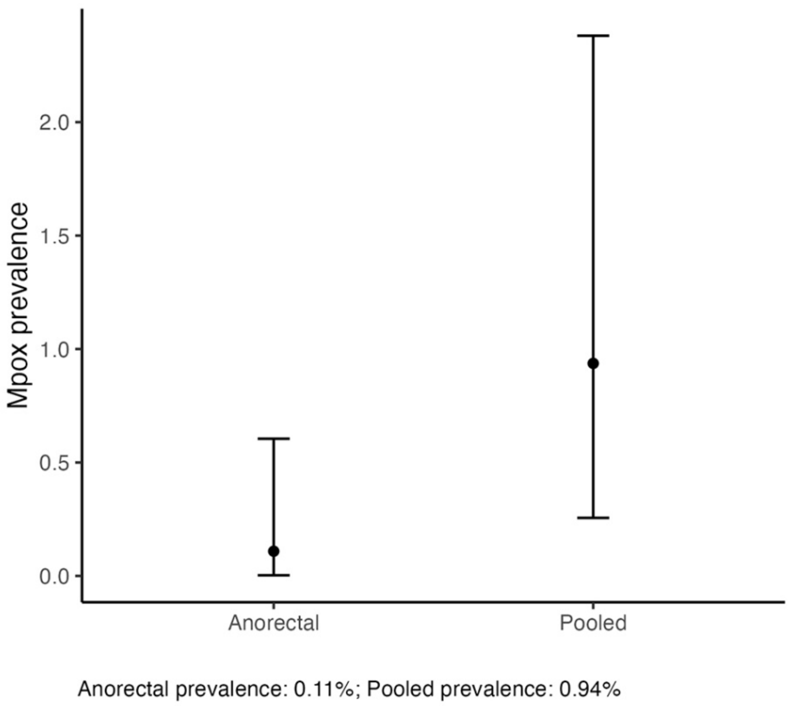


**Appendix Figure 1.** Whisker plot of mpox prevalence by testing modality in a study of prevalence of asymptomatic mpox among men who have sex with men, Japan, January–March 2023. Samples were self-collected by study participants. Pooled samples consisted of anorectal swabs, initial stream urine, and gargle rinse. Bars indicate 95% CI; dots indicate median.

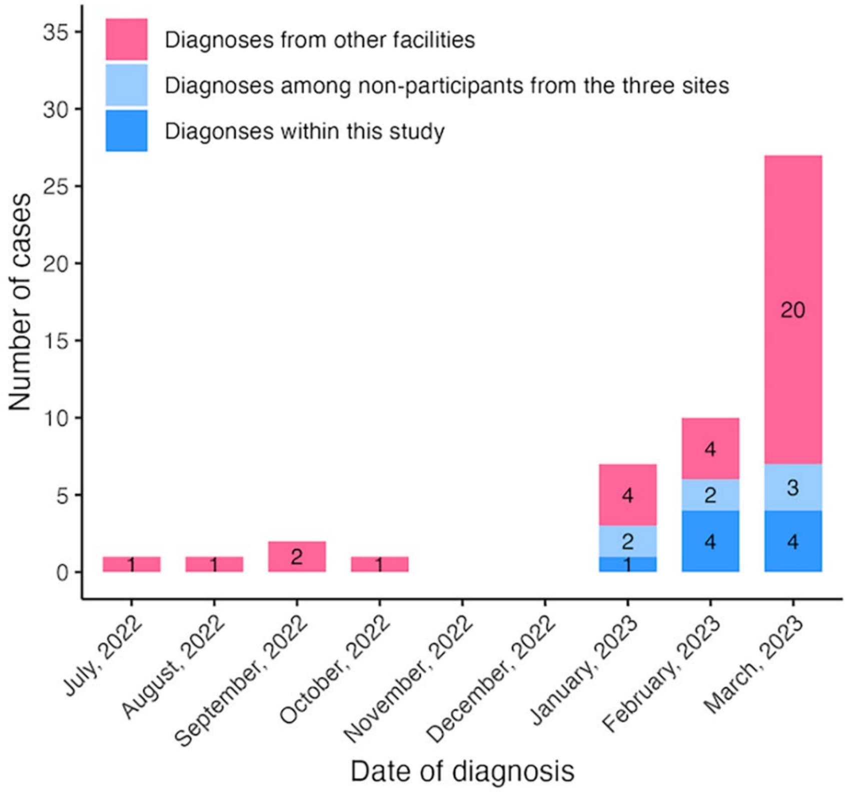


**Appendix Figure 2.** Number of mpox infections in Tokyo July 2022–March 20, 2023, in a study of prevalence of asymptomatic mpox among men who have sex with men, Japan. A total of 49 mpox cases had been reported in Tokyo as of March 20, 2023; 44 were reported during the study period. The stacked bar chart shows the breakdown of cases reported from participants in this study, cases reported outside the study but from the same study sites, and cases reported from facilities not included in the study.
